# Improving mental health through body-awareness with dynamic interpersonal therapy in patients with persistent somatic symptoms: an explorative cohort study

**DOI:** 10.3389/fpsyt.2026.1661471

**Published:** 2026-02-18

**Authors:** Jordy Rovers, Sandra Braam, Mia Scheffers, Jackie Scharroo

**Affiliations:** 1 Department of Psychiatry, specialized treatment centre for persistent physical symptoms, Canisius-Wilhelmina Hospital, Nijmegen, Netherlands; 2 Department of Psychiatry, Radboudumc, Nijmegen, Netherlands; 3 School of Human Movement and Education, Windesheim University of Applied Sciences, Zwolle, Netherlands

**Keywords:** body awareness, mental health, persistent somatic symptoms, psychodynamic psychotherapy, somatic symptom disorder

## Abstract

**Introduction:**

This study explored the changes in mental health and body-awareness in patients with severe persistent somatic symptoms (PSS) treated with multidisciplinary Dynamic Interpersonal Therapy.

**Methods:**

In this longitudinal study 56 patients with severe somatic-symptom disorder (DSM-5) were included. Analyses were conducted on available outcome data from 32–38 patients. All were treated with a multidisciplinary DIT program for 6 months in a specialized care facility. Patients were followed up during treatment with the questionnaires Mental Health Continuum Short Form (MHC-SF) and Multidimensional Assessment of Interoceptive Awareness (MAIA) at baseline, 3 months and 6 months. Change was analysed with repeated measures ANOVA. The association between change in MAIA and change in MHC-SF was explored with linear regression analysis.

**Results:**

Both mental health (MHC-SF total score 28 to 33, p<0.001) and interoceptive awareness (MAIA score 88 to 92 p=0.045) increased after treatment. The improvement was mostly seen in the psychological and social wellbeing subscales of the MHC-SF and the self-regulation and body listening subscales of the MAIA. A higher pre- to post-treatment change on the MAIA was associated with a higher change on the MHC-SF (R2 = 0.352, B = 1.464, p < 0.001).

**Conclusions:**

This study shows that patients with PSS 1 have lower mental health scores relative to general population norm, 2) seem to have reduced capacity for interoceptive awareness 3) improve in both areas after completing DIT for PSS and 4) shows that improvement in interoceptive awareness was associated with improvement in mental health. Interoception based interventions in DIT-PSS might be a starting point for adequate treatment of PSS.

## 1 Introduction

With a an estimated 4% prevalence rate in the general population, somatic symptom disorder (SSD) is one of the most common mental disorders as described in the DSM-5 (1–3). Prevalence rates are likely higher in primary and secondary health care settings; however, recent and methodologically comparable epidemiological studies using contemporary SSD criteria are currently lacking. Patients with SSD suffer from bodily symptoms that persist for at least 6 months which disrupt their daily life and are accompanied by excessive or disproportionate thoughts, feelings, or behaviours related to the symptoms (1). SSD often comes accompanied with severe suffering, lowered mental health and comorbid diagnoses such as major depressive disorder, anxiety disorders and personality disorders (2, 4–6). Persistent somatic complaints may also be classified under other diagnostic frameworks, including somatoform disorders (ICD-10), bodily distress disorder (ICD-11), and single functional somatic syndromes such as irritable bowel syndrome, fibromyalgia, functional neurological disorder, and chronic fatigue syndrome.) (7).

Across these classifications, symptoms are often only partially explained by identifiable biomedical pathology. Contemporary models therefore conceptualize these complaints as the result of an interplay between biological vulnerability, altered neurophysiological processing, psychological factors, and social context (8). Central mechanisms such as altered interoceptive processing, heightened symptom-focused attention, and impaired integration of bodily sensations into coherent mental representations are increasingly recognized as key factors underlying symptom persistence (2, 8–14). The overarching term of Persistent Somatic Symptoms (PSS) is used to describe all of the distressing somatic complaints, irrespective of their aetiology, that are present on most days for at least several months, and in which biological, psychological and social factors play a role in maintaining these somatic complaints (11).

Treatment options for PSS range from patient and family management, pharmacological interventions, such as antidepressants, to extensive psychological or somatic treatment (15–17). The choice of treatment is dependent on the severity, nature and duration since the onset of the symptoms (3). Psychotherapy, such as cognitive behavioral therapy is found to be effective in patients with PSS and is advised in current guidelines (18, 19). When first line psychological or physical therapies fail, intensified treatment in specialized multidisciplinary facilities is advised (3, 17). Yet, more evidence into the effectiveness and working mechanism of psychotherapies, integrated treatment modalities or physical therapies is needed (18).

Body- and movement-oriented therapies, such as psychosomatic physical therapy and psychomotor therapy, are often applied simultaneously with other more verbally oriented psychological interventions. This combined treatment often takes place in specialized treatment facilities. Body- and movement-oriented therapies aim to enhance interoceptive awareness (20). Interoception refers to the processing of internal bodily stimuli by the nervous system which in turn drives our behavior, cognitions and emotions. Dysfunctional interoceptive awareness is increasingly recognized as an important component in mental health disorders (9). It has been observed that interoceptive awareness is low in patients with SSD (10, 13). By intervening on this specific underlying problem, people might regain more interoceptive awareness and this might in turn heighten mental health. However, the relationship between interoceptive awareness and mental health changes due to clinical interventions is as yet unknown and therefore the main focus of this study.

At the Canisius Wilhelmina Hospital (CWZ) in Nijmegen, the Netherlands, a multidisciplinary treatment according to the principles of Dynamic Interpersonal Therapy (DIT), adjusted for PSS, is offered to severely impaired patients with SSD (14, 21). Practically all of these patients showed limited to no effect on earlier stand-alone treatments, such as Cognitive Behavior Therapy (CBT) or physical therapy. They are enrolled in a twice weekly, six months long, day-treatment in which group DIT psychotherapy, psychosomatic physical therapy, psychomotor therapy, art therapy, and behavioral therapy are integrated (22).

DIT was selected as the core psychotherapeutic framework for this study because of its focus on affect regulation, interpersonal patterns, and mentalization, all of which are considered central to the maintenance of PSS (14). Within psychodynamic models, bodily symptoms are conceptualized as expressions of non-mentalized affect and difficulties in integrating bodily sensations into meaningful psychological representations. For patients with PSS, DIT was specifically adapted to include a stronger emphasis on bodily experience and interoceptive awareness, and was embedded in a multidisciplinary treatment program combining verbal psychotherapeutic interventions with body- and movement-oriented therapies (21). This integration aims to facilitate bodily mentalization and to support patients in linking bodily sensations to emotional and interpersonal experiences within a safe therapeutic context (22).

We hypothesize that patients with PSS 1) show substantially lower mental health scores compared with general population norms, 2) demonstrate relatively limited interoceptive awareness, 3) show improvement in both areas after completing the program and 4) show a positive association between changes in interoceptive awareness and changes in mental health.

## 2 Materials and methods

### 2.1 Study population and design

Patients that received treatment for PSS and were classified as suffering from somatic-symptom disorder according to the DSM 5 between June 2018 and June 2019 at the Canisius Wilhelmina Hospital (CWZ) in Nijmegen, the Netherlands, were asked to participate in this study. Patients referred to the specialized treatment centre typically had a history of persistent and severely impairing somatic symptoms and had often previously received first-line treatments, such as cognitive behavioural therapy or physical therapy, without sufficient or sustained improvement. The multidisciplinary DIT-based program described below constituted the study intervention. In this period 63 patients received treatment at our facility and all were asked to participate. This study had a prospective observational cohort design.

### 2.2 Treatment procedure

Patients (*≥* 18 years) with often long-standing, severely impairing, persistent medical symptoms were referred for treatment by general practitioners, medical specialists or registered mental healthcare providers. All patients were assessed by a psychiatrist, clinical psychologist or psychotherapist. In this assessment psychiatric examination took place as well as an indication setting for treatment when SSD was diagnosed. When there was a history of psychosis, a psychotic personality structure, severe drug abuse, an IQ below 80, a primary eating disorder with a BMI < 17.5 or when the medical symptoms were inadequately examined by prior healthcare professionals, patients were not found suited for treatment at our facility and other treatments or further examination was advised.

### 2.3 Intervention

The treatment offered is Dynamic Interpersonal Therapy (DIT), adjusted for patients with PSS (14). DIT focusses on improving the capacity to (bodily) mentalize and changing stressful interpersonal patterns by combining interventions originating from psychodynamic psychotherapy theorems. In our treatment facility it is offered as a multidisciplinary group treatment consisting of DIT psychotherapy, psychosomatic physical therapy, psychomotor therapy, art therapy and behavioural therapy. The complete intended treatment takes six months, twice a week. The group consists of a maximum of eight patients. After three months half of the group ends therapy and new patients start, making it a semi-open group design. For more details we refer to the treatment protocol as described by Selders et al. and more recently by Maatkamp et al. (14, 22).

### 2.4 Measures

The outcome measures were structurally collected on paper by SB at the start of treatment (baseline), at 3 months and at the end of treatment at 6 months.

#### 2.4.1 Mental health continuum – short form

The primary outcome of this study was mental health, measured through the MHC-SF, which is a validated questionnaire in the Netherlands (23). The MHC-SF consists of 14 questions about a range of mental health issues. All questions refer to the past month. Questions are scored on a 6-point Likert scale. There a three subscales: emotional wellbeing (questions 1 to 3), social wellbeing (4 to 8) and psychological wellbeing (9 to 14). A higher score indicates a better mental health with a maximum score of 70. There are no cut-off scores available, however the mean score in a general population in the Netherlands was 45 (24).

#### 2.4.2 Multidimensional assessment of interoceptive awareness

The MAIA measures general, non-situational awareness of the body (25) and was the secondary outcome. The questionnaire consists of 32 statements. The respondent is asked to indicate on a 6-point scale (0 = not applicable; 5 = fully applicable) to what extent the statement applies. There are eight subscales: Noticing (4 items), Not-distracting (3 items), Not-worrying (3 items), Attention regulation (7 items), Emotional awareness (5 items), Self-regulation (4 items), Listening to the body (3 items), and Trust of the body (3 items) (Mehling et al., 2012). The total score ranges from 0 to 160, A higher score means a better awareness of the body, yet there are no cut-off scores available The MAIA has been investigated for validity, reliability and psychometric characteristics (26).

### 2.5 Statistical analysis

Data was analysed using IBM software SPSS version 27. Demographic statistics were performed on all participants. We performed repeated measures ANOVA to compare pre-, during and post-treatment measurements. We calculated the estimated effect size using Cohen’s d. With linear regression analysis we examined whether the percentage of change in mental health was influenced by the percentage of change in interoceptive awareness with age and sex as covariates.

### 2.6 Ethics

This study was reviewed by the medical-ethical committee [(Commissie Mensgebonden Onderzoek (CMO)] region Arnhem-Nijmegen under file-number 2018-4096. The committee concluded that the study does not involve procedures or behaviours that fall under the scope of the Medical Research Involving Human Subjects Act (WMO). Therefore, formal approval by a recognized Medical Ethics Review Committee was not required. In addition, the local ethics committee and the Board of Directors of the CWZ reviewed and approved the study for execution. All participants provided written informed consent prior to participation. The study was conducted in accordance with the Declaration of Helsinki and the principles of Good Clinical Practice (GCP).

## 3 Results

Of the 63 patients that were approached, 56 decided to participate and gave informed consent. 17 participants were male (30%) and the mean age was 45.5 [SD 12.4]. 38 participants had some measures available at the end of the study period, only 35 had complete pre-, during and post measures and completed the treatment. These patients were included in the analyses.

### 3.1 Mental health

The mean total scores of the MHC-SF pre-, during and post-treatment are significantly different (F = 7.5, p = 0.001). The overall mental health is scored significantly higher after treatment than before treatment, indicating an increase of overall mental health throughout the course of treatment with a medium effect size (Cohen’s d = 0.67). See **Table 1**.

**Table 1:** Mental health outcomes.

Measures	N	Baseline	3 months	6 months	p-value*	Cohen’s d**
*Mental health* *(MHC-SF total)*	*35*	*28.4 [11.5]*	*29.5 [13.7]*	*33.2 [13.7]*	*0.001*	*0.67*
*Emotional wellbeing* *(MHC-SF 1-3)*	*36*	*6.4 [3.7]*	*6.4 [3.4]*	*7.1 [3.5]*	*0.058*	*-*
*Social wellbeing* *(MHC-SF 4-8)*	*37*	*8.6 [4.2]*	*8.7 [5]*	*10.2 [4.5]*	*0.011*	*0.52*
*Psychological wellbeing* *(MHC-SF 9-14)*	*38*	*12.5 [5.8]*	*13.6 [6.4]*	*15.8 [6.9]*	*0.002*	*0.58*

data are means [SD]. *calculated by repeated measures ANOVA. **effect-size for pre- to post-treatment effect based on paired sample t-test, only for significant pre–post differences.

#### 3.1.1 Emotional wellbeing

The mean sum score on emotional wellbeing is not significantly different between the three time points (F = 3, p = 0.058). There seems to be no significant change in emotional wellbeing throughout the course of treatment.

#### 3.1.2 Social wellbeing

The mean sum score on social wellbeing is significantly different between the three time points (F = 4.9, p = 0.011), indicating an increase in social wellbeing throughout the course of treatment.

#### 3.1.3 Psychological wellbeing

The mean sum score on psychological wellbeing is significantly different between the three time points (F = 7.9, p = 0.002). Again, the post-treatment score is higher than the pre-treatment score, indicating an increase in psychological wellbeing throughout the course of treatment.

### 3.2 Interoceptive awareness

The positive change in interoceptive awareness as measured through the total score of the MAIA from pre-, during and post-treatment shows statistical significance (F = 3.3, p = 0.045) (see **Table 2**). The subscales ‘‘noticing’’, ‘‘not-worrying’’, ‘‘attention regulation’’, ‘‘emotional awareness’’ and ‘‘trusting’’ show no significant change. The subscales ‘‘self-regulation’’ and ‘‘body listening’’ however do show significant change with higher scores post-treatment and ‘‘not-distracting’’ with lower post-treatment scores.

**Table 2:** Interoceptive awareness outcomes.

Measures	N	Baseline	3 months	6 months	p-value*
Interoceptive awareness (MAIA total)	32	87.8 [16]	84.5 [19]	91.7 [17]	0.045**
Noticing	37	3.4 [0.79]	3.2 [0.98]	3.3 [0.72]	0.239
Not-distracting	34	3.7 [0.84]	3.7 [0.78]	3.3 [0.82]	0.014**
Not-worrying	34	2.7 [0.95]	2.5 [1.0]	2.5 [0.84]	0.176
Attention regulation	33	2.3[0.78]	2.3 [0.84]	2.5 [0.83]	0.240
Emotional awareness	34	3.4 [1.1]]	3.2 [0.97]	3.5 [0.77]	0.183
Self-regulation	32	2.1 [0.89]	2.0 [0.91]	2.5 [1.01]	0.032**
Body Listening	33	1.9 [1.30]	2.2 [1.10]	2.6 [0.91]	0.003**
Trusting	33	2.3 [1.23]	2.0 [1.20]	2.4 [1.16]	0.134

data are means [SD]. *calculated by repeated measures ANOVA. ** significant change. Effect sizes were not calculated for MAIA subscales, given the exploratory nature of the analyses and the use of repeated measures ANOVA across three time points.

### 3.3 Association of change in interoceptive awareness with change in mental health

The mean percentage of change on the MHC-SF pre to post treatment is 12% [SD = 45]. There is an positive association between change in interoceptive awareness as measured by the MAIA and change in mental health. Linear regression analysis showed that a higher percentage of change on the MAIA was significantly associated with a higher change on the MHC-SF with an explained variance of 35% (R^2^ = 0.352, B = 1.464, p < 0.001) when corrected for age and sex.

## 4 Discussion

This naturalistic study shows that patients with PSS might 1) have lower mental health scores relative to general population norm, 2) seem to have reduced capacity for interoceptive awareness, 3) improve in both areas after completing DIT for PSS and 4) shows that improvement in interoceptive awareness were associated with improvement in mental health. The first three results are in line with existing literature about PSS patient characteristics and the effectiveness of DIT for PSS (10, 21). They are also in accordance with the findings of a recent systematic review showing that interoception based interventions are efficacious for improving interoceptive awareness across multiple mental health disorders and specifically in PSS disorders (27). This in turn supports the described common therapeutic strategies utilized in the treatment of PSS, which often include interventions aimed at improving interoceptive awareness (12). Our study expands the current knowledge showing the potential effect of DIT-PSS on interoceptive awareness and its positive association with change in mental health. This is, as far as we know, the first naturalistic study to investigate the theory of improvement of interoceptive awareness as a possible starting point in the treatment for PSS in clinical practice, using a psychodynamic approach such as DIT-PSS.

We found an effect of DIT-PPS on the MAIA subscales ‘‘self-regulation’’, ‘‘body listening’’ and ‘‘not-distracting’’. Klocek et al. also found that ‘‘self-regulation’’ and ‘‘body listening’’ improved and contributed indirectly to well-being for patients treated with psychodynamic psychotherapy for different disorders (28). De Jong et al. found that Mindfulness Based Cognitive Therapy resulted in positive changes on ‘‘self-regulation’’ and ‘‘not-distracting’’ for patients with chronic pain and depression (29). As was also the case in these studies, we did not find changes on the other MAIA subscales. It might be that these changes are not yet present directly after treatment and take more time. This is subject for further research with at least a longer follow-up after treatment. Although quantitative changes were modest, it is important to note that changes in mental health and interoceptive functioning may be difficult to fully capture with questionnaires alone. In earlier qualitative work within the same treatment program, patients described shifts in bodily awareness, emotional understanding, and interpersonal functioning that unfolded gradually over time (22). These qualitative findings may help contextualize the present results and suggest that the observed quantitative changes reflect only part of a broader therapeutic process. From this perspective, therapeutic change may involve both increased tolerance of bodily sensations within a safe therapeutic context and a gradual transformation in the mental representation and meaning of bodily processes, as has also been discussed in qualitative psychoanalytic work (30). However, these processes cannot be disentangled within the present study design.

### 4.1 Limitations & strengths

However promising our results may be, they should be interpreted with some caution. First, our study has a non-randomized, uncontrolled design. The absence of a control group reflects the exploratory and naturalistic aim of this study, which was to examine associations between interoceptive awareness and mental health within routine clinical practice rather than to establish treatment efficacy. Thus, it is unknown whether the results are due to the therapy program alone. Yet we treat a subgroup of patients with severe PSS that have not responded to first-line treatments which makes it unlikely that they would improve without multidisciplinary treatment (31, 32). Second, we have a small sample of 56 patients of which only 38 remained at the end of treatment. This is a drop-out rate of 32%, which is comparable to the 20-60% drop-out rates for psychotherapy mentioned in literature, yet higher than the drop-out for psychodynamic group therapy (20.6%) reported in literature (33, 34). This may have influenced our results, since the drop-outs might be those patients not benefiting from DIT or those not keen on participating in this study any longer due to experienced high levels of burden. In addition, although statistically significant, the absolute changes in mental health and interoceptive awareness were modest. This may reflect the severity and chronicity of the studied SSD population, in whom large short-term changes on broad, dimensional outcome measures are uncommon. Third, the treatment program of DIT for PSS has a multidisciplinary approach with different treatment modalities, making it impossible to study the effect of each modality separately. We therefore can only interpret our results as a possible effect of the complete treatment protocol of DIT for PSS. It might therefore also be the case that a positive change in mental health might lead to improved interoceptive awareness, but literature is lacking. For future research it could therefore be needed to study the contributing factors of individual interventions, for example by a randomized group comparison of DIT psychotherapy alone and DIT psychotherapy with interoception based interventions (e.g. physical therapy, mindfulness, psychomotor therapy).

The strength of this study lies in the clinical and naturalistic design, making it one of the first studies to investigate the relationship of interoceptive awareness and mental health in multimodal psychodynamic psychotherapy for PSS (DIT-PSS). For future research the effect of interoception based interventions should be explored further in both quantitative and qualitative designs. Specifically qualitative studies might shed light on the underlying working mechanisms of interventions targeting interoceptive awareness as well as on the experience of physical symptoms and mental health. In turn this may enhance the current practice of treatment of PSS.

## 5 Conclusion

This clinical study suggests that improvement in interoceptive awareness might lead to more beneficial outcome in mental health. Therefore, more research into interception based interventions might be a starting point to find more adequate treatment options of severe and difficult to treat PSS. Further clinical quantitative as well as qualitative studies into the understanding of this association might enhance treatment for PSS.

## Data Availability

The raw data supporting the conclusions of this article will be made available by the authors, without undue reservation.
